# Supplementary material for: Leaf Transcriptome Analysis of Broomcorn Millet Uncovers Key Genes and Pathways in Response to Sporisorium destruens
Source: Int J Mol Sci. 2021 Sep 2;22(17):9542. doi: 10.3390/ijms22179542 (PMC8430493; doi:10.3390/ijms22179542)
Supplement: Supplementary file 1 [file ijms-22-09542-s001.zip › Supplementary Materials file 1.pdf]

Supplementary Materials file S1 Evaluation of sequencing quality and Statistics of mapping efficiency

**Sample ID**	Clean bases	GC content(%)	Q30(%)	Total Reads	Total mapped	Multiple mapped	Uniquely mapped
**R0_1**	7394648932	55.11	95.71	49687466	48076562(96.76%)	3206544(6.45%)	44870018(90.3%)
**R0_2**	7881934621	54.52	95.75	53211570	51079283(95.99%)	2437425(4.58%)	48641858(91.41%)
**R0_3**	7312145290	54.49	95.55	49209774	47540743(96.61%)	2665151(5.42%)	44875592(91.19%)
**R1_1**	7299695007	54.14	95.76	49000544	47314648(96.56%)	2903289(5.93%)	44411359(90.63%)
**R1_2**	6497644579	54.36	95.64	43519658	40731898(93.59%)	2124823(4.88%)	38607075(88.71%)
**R1_3**	7797746941	54.11	95.87	52434762	50791279(96.87%)	2711374(5.17%)	48079905(91.69%)
**S0_1**	7124815466	54.09	95.48	47918608	46290057(96.6%)	2557405(5.34%)	43732652(91.26%)
**S0_2**	7886489958	53.93	95.77	52846108	51325589(97.12%)	2615927(4.95%)	48709662(92.17%)
**S0_3**	7331779998	53.90	95.58	49215266	47731377(96.98%)	2826175(5.74%)	44905202(91.24%)
**S1_1**	7155568210	54.78	95.51	48065402	46501341(96.75%)	2910804(6.06%)	43590537(90.69%)
**S1_2**	7276884690	54.42	95.75	48884800	47231250(96.62%)	3297921(6.75%)	43933329(89.87%)
**S1_3**	7350768099	55.02	95.51	49377350	47618959(96.44%)	2958022(5.99%)	44660937(90.45%)
**S2_1**	7283244877	54.17	96.07	48970932	47383138(96.76%)	3629590(7.41%)	43753548(89.35%)
**S2_2**	7692810160	54.56	95.81	51646700	50033277(96.88%)	2915025(5.64%)	47118252(91.23%)
**S2_3**	7075754640	54.63	95.47	47481276	45967525(96.81%)	3676372(7.74%)	42291153(89.07%)
