# Supplementary material for: Leaf Transcriptome Analysis of Broomcorn Millet Uncovers Key Genes and Pathways in Response to Sporisorium destruens
Source: Int J Mol Sci. 2021 Sep 2;22(17):9542. doi: 10.3390/ijms22179542 (PMC8430493; doi:10.3390/ijms22179542)
Supplement: Supplementary file 1 [file ijms-22-09542-s001.zip › Supplementary Materials file 8.pdf]

Supplementary Materials file S8. List of primer used for RT-PCR analysis

Gene identifier	Primer (5' to 3')	Product size (bp)
Longmi016403	F: AGCAACACCTCGGCTGATTAC<br>R: CACCCGCTGCGATGAACT	1074
Longmi012456	F: GGCTTCAACGACTTCTCC<br>R: TGTTCACGGCTTCCACTT	1449
Longmi040491	F: CCCATACATTTGTGGAAGGC<br>R: AGAGGCGTGGATGGCAGT	1161
Longmi034630	F: TGACACCGTCGATCTCGT<br>R: GCACCTCTGGGTTAGGAA	1023
Longmi035893	F: GGCAAGATGGTGGTGTTC<br>R: TCTGTGAGGCTGGGCTGT	1180
Longmi057592	F: CCGCACTTCCGCTACACC<br>R: CCAGACCATGAGCCCGTC	1098
Longmi046299	F: ATGAACCGCCAGAACGAGG<br>R: CTGAACTGCCCGATGAGGA	1209
Longmi029722	F: GACGACATCGGCAACACCACC<br>R: CCAGCAGCAGGAACTTGAGCAG	441
Longmi052503	F: GGAGGAGAAGAGGGGGAAGG<br>R: GGAAGAGCGGGTGGTAGAGG	351
Longmi013362	F: GGGCTGAAGCTGGAGTCCCG<br>R: GATGCCGACGCCGATGATGT	414
actin	F: ACCGAAGCCCCTCTTAACCC<br>R: GTATGGCTGACACCATCACC	158
